# Subcellular localization of PUMA regulates its pro-apoptotic activity in Burkitt's lymphoma B cells

**DOI:** 10.18632/oncotarget.5901

**Published:** 2015-09-29

**Authors:** Gorbatchev Ambroise, Alain Portier, Nathalie Roders, Damien Arnoult, Aimé Vazquez

**Affiliations:** ^1^ INSERM, UMR_S 1197, Hôpital Paul Brousse, Villejuif, France; ^2^ Université Paris-Saclay, France; ^3^ Equipe Labellisée Ligue contre le Cancer, Villejuif, France

**Keywords:** PUMA, Burkitt's Lymphoma, apoptosis, translocation, mitochondria

## Abstract

The BH3-only protein PUMA (p53-upregulated modulator of apoptosis) is a major regulator of apoptosis. It belongs to the Bcl-2 family of proteins responsible for maintaining mitochondrial outer membrane integrity by controlling the intrinsic (mitochondrial) apoptotic pathway. We describe here a new pathway regulating PUMA activation through the control of its subcellular distribution. Surprisingly, neither PUMA upregulation in normal activated human B lymphocytes nor high levels of PUMA in Burkitt's lymphoma (BL) were associated with cell death. We show that PUMA is localized to the cytosol in these cells. By contrast, various apoptosis-triggering signals were found to promote the translocation of PUMA to the mitochondria in these cells, leading to their death by apoptosis. This apoptosis was associated with the binding of mitochondrial PUMA to anti-apoptotic members of the Bcl-2 family, such as Bcl-2 and Mcl-1. This translocation was caspase-independent but was prevented by inhibiting or knocking down the expression of the MAPK kinase p38. Our data suggest that the accumulation of PUMA in the cytosol may be important for the participation of this protein in apoptosis without the need for prior transcription. This regulatory pathway may be an important feature of differentiation and tumorigenic processes.

## INTRODUCTION

Apoptosis is regulated by two major groups of proteins: caspases and the proteins of the Bcl-2 family. Bcl-2 family proteins regulate the integrity of the mitochondrial outer membrane (MOM) and can be classified into three different groups: pro-apoptotic, anti-apoptotic and BH3-only regulators. The pro-apoptotic molecules Bax and Bak are directly responsible for the formation of pores in the MOM, allowing the exit of apoptogenic factors, such as cytochrome-*c*. The anti-apoptotic molecules, such as Bcl-2, Bcl-xl, Bcl-w, A1 or Mcl-1, can inhibit Bax/Bak-dependent MOM permeabilization (MOMP). They can be classified into two subgroups, Mcl-1/A1 and Bcl-2/Bclxl/Bclw, according to their sensitivity to BH3-only proteins and to various inhibitors, such as ABT-737. The third group, BH3-only proteins, contains proteins regulating the other two groups and controlling the pro/antiapoptotic balance. This group can also be classified into two subgroups: (i) BH3-only proteins binding only one subgroup of anti-apoptotic molecules (e.g. Bad and Bik interact with and inhibit the Bcl-2 subgroup whereas Noxa and Bmf interact with the Mcl-1 subgroup) and (ii) BH3-only proteins able to interact with all anti-apoptotic molecules, such as tBid, Bim and PUMA. These two subgroups also differ in their capacity to activate pro-apoptotic proteins directly. Only the proteins of the second subgroup (tBid, Bim and PUMA) have been reported to interact with and activate Bax and Bak directly [1-3].

PUMA (p53-upregulated modulator of apoptosis), also known as Bbc3, was described simultaneously by several different groups [4] [5]. Its gene encodes four different isoforms (α, β, γ and δ). Only, the α (23 kDa) and β (18 kDa) isoforms have apoptotic properties. Both these isoforms contain the BH3 domain, essential for homotypic interaction with other Bcl-2 family members, and the C-terminal domain, which includes a putative transmembrane domain [4]. PUMA gene transcription is regulated by p53-dependent and p53-independent pathways including various transcription factors, such as Foxo3A, C/EBP and E2F1 [4, 6-8]. PUMA can promote apoptosis by either direct interactions with and inhibition of all anti-apoptotic molecules or the direct activation of Bax and Bak, leading to MOMP [4, 9]. Triple-KO mice (Bid/Bim/PUMA) have the same phenotype as Bax/Bak KO mice, whereas Bim/Bid double-KO mice do not, highlighting the importance of PUMA in the mitochondrial apoptotic pathway [10]. PUMA has been implicated in various apoptotic responses, including DNA damage, growth factor/cytokine withdrawal and the response to glucocorticoids[11, 12] [13]. PUMA has also recently been shown to play a major role in the control of memory T- and B-lymphocyte survival [14, 15]. In this context, activated B cells produce large amounts of PUMA, which is associated with cell activation rather than cell death [15]. This raises questions about the mechanisms preventing PUMA-induced apoptosis in activated lymphocytes. We show here that this paradoxical situation is due to localization of PUMA to the cytosol in these cells. We also show that, in response to apoptotic stimuli, PUMA is translocated from the cytosol to the mitochondria, where it can bind anti-apoptotic molecules, including Bcl-2 and Mcl-1, leading to cell death.

## RESULTS

### PUMA is present in the cytosol of activated B cells

**Figure 1:**
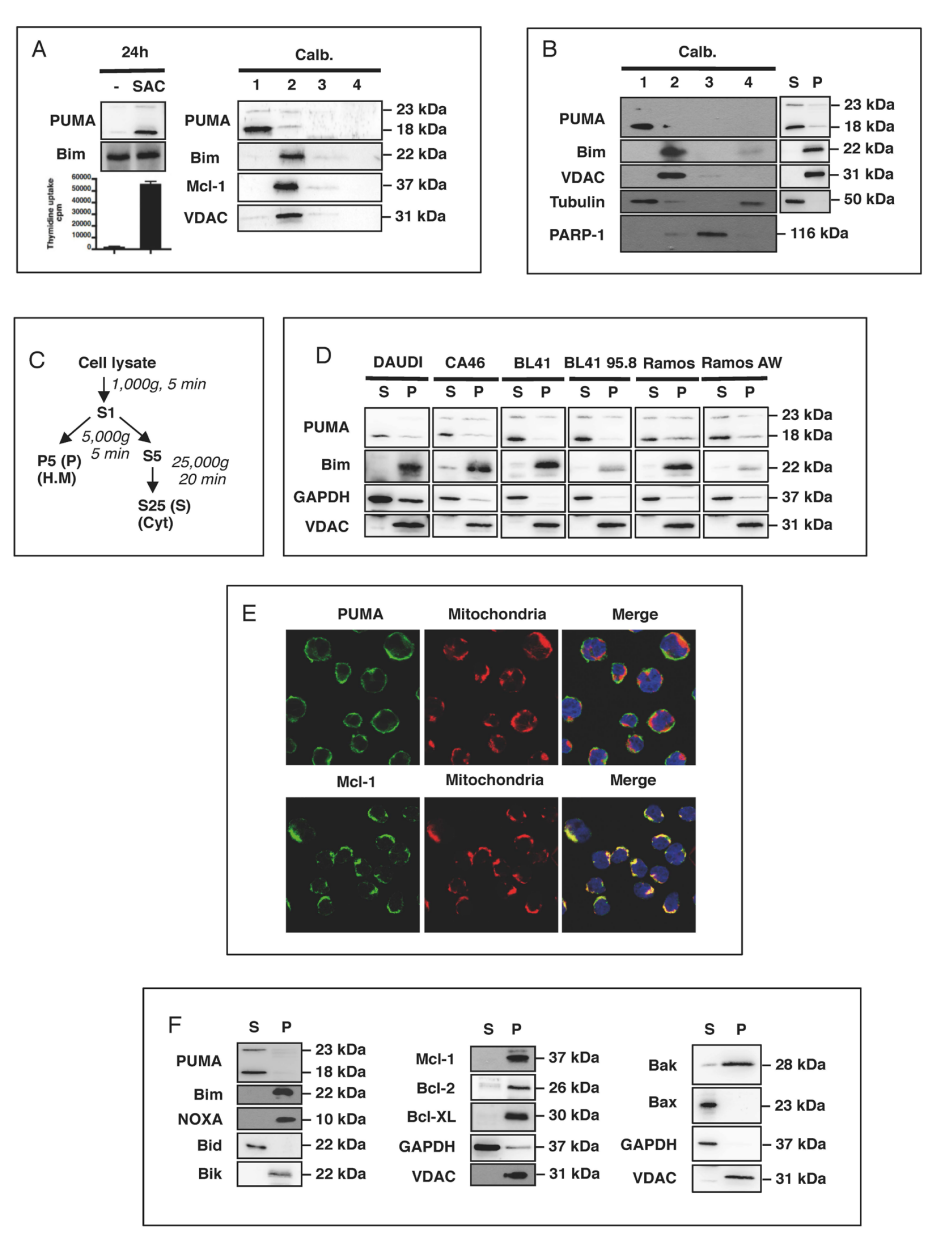
PUMA is present in the cytosol of activated B cells **A.** Human tonsilar B cells were treated with mitogenic doses of *Staphylococcus aureus* strain Cowan 1 (pansorbin: SAC) at a dilution of 1/10,000 for 24 h. Proliferation was assessed by measuring DNA synthesis, as assessed from ^3^H-thymidine incorporation during the last 16 h of culture, in counts per minute (cpm). Cells were fractionated with the Calbiochem^®^ extraction kit and western blotting was used to determine the subcellular localization of PUMA, Bim and Mcl-1 in the fractions (1: cytosol; 2 heavy membrane; 3: nucleus and 4: less soluble material associated with the cytoskeleton). **B.** Resting BL41 cells were fractionated as in **A.** and the subcellular localization of PUMA, Bim, VDAC, tubulin and PARP was assessed by western blotting. **C.** Purification of the cytosol (S) and heavy membrane (P) fractions by differential centrifugation of cell lysates. **D.** Cell lysates from DAUDI, CA46, BL41 and Ramos Burkitt's lymphoma cell lines and their EBV-positive counterparts (BL41 95.8 for BL41 and RamosAW for Ramos) were fractionated and the S and P fractions tested for PUMA, Bim, GAPDH and VDAC by western blotting. **E.** BL41 cells were stained with anti-PUMA, anti-Mcl-1 and anti-TOM20 primary antibodies with their corresponding fluorochrome-conjugated secondary antibodies, green for PUMA and Mcl-1 or red for TOM20, and the subcellular distribution of PUMA and Mcl-1 was analyzed by confocal microscopy (E, right panel). **F.** Resting BL41 cells were fractionated as shown in **C.** and the subcellular distributions of the various Bcl-2 family members were analyzed by western blotting.

**Figure 2:**
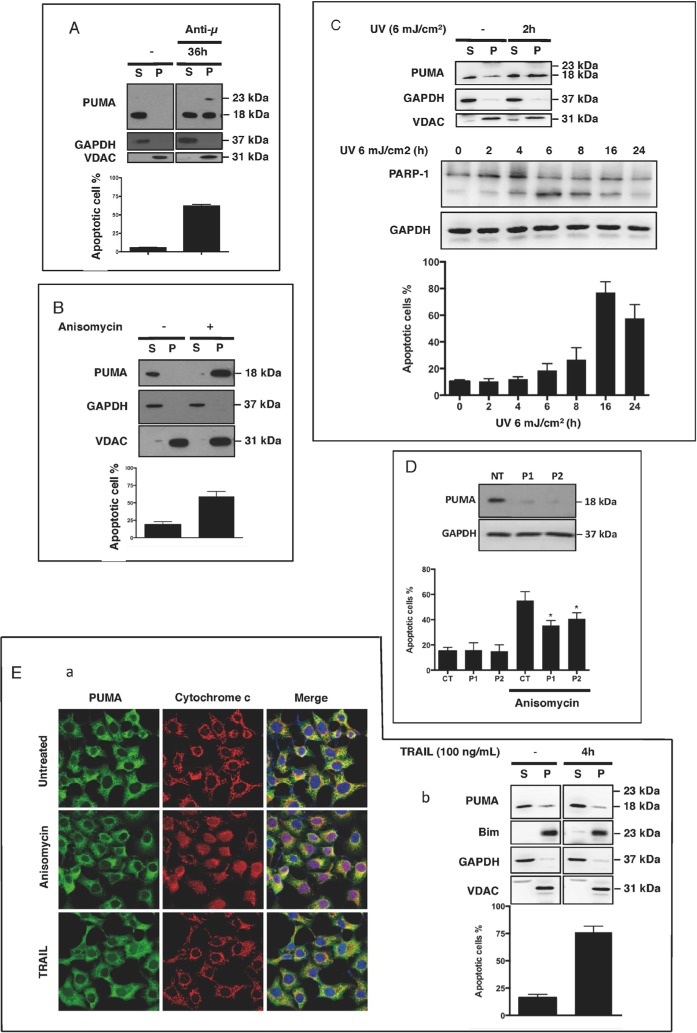
PUMA is found at the mitochondria when apoptosis is triggered **A.** BL41 cells were stimulated for 36 h with mouse anti-human μ antibodies (5 μg/ml) cross-linked with anti-mouse IgM antibodies (28 μg/ml). Western blotting was used to test the cytosol (S) and heavy membrane fractions (P) for PUMA. Apoptosis was assessed by flow cytometry and cells were considered apoptotic if they were shrunken, with high side scatter and low forward scatter. The data shown are means ± SD for triplicate experiments. **B.** BL41 cells were stimulated with anisomycin (2 μg/ml) for 4 h. The S and P fractions were tested for PUMA, GAPDH and VDAC by western blotting, and apoptosis was assessed by flow cytometry. **C.**. HeLa cells were exposed to UV (6 mJ/cm^2^) and cultured for 2 h. Cell lysates were fractionated and the fractions were tested for PUMA, GAPDH and VDAC by western blotting. Cell shrinking and PARP-1 cleavage were assessed by flow cytometry and western blotting, respectively, at the indicated times following UV exposure. The data shown are means ± SD for triplicate experiments **D.** BL41 cells transfected with a non-targeting siRNA (CT) or a PUMA-targeting siRNA (P1 and P2) for 76 h were treated for 4 h with anisomycin (2 μg/ml) or left untreated (controls). Apoptosis was analyzed by flow cytometry (means ± SD for triplicate experiments) and PUMA knockdown efficiency was analyzed by western blotting with GAPDH as a loading control. **E.** HeLa cells were or were not activated with recombinant TRAIL (100 ng/ml) for 4 h. (panel a) Cells were stained with anti-PUMA and anti-cytochrome *c* primary antibodies with their corresponding fluorochrome-conjugated secondary antibodies, green for PUMA and red for cytochrome *c*, and the subcellular localization of PUMA and cytochrome *c* was analyzed by confocal microscopy. (panel b) S and P fractions were tested for PUMA, Bim, GAPDH and VDAC by western blotting, and apoptosis was assessed by flow cytometry.

We previously observed that PUMA expression was strongly upregulated in response to mitogenic activation *in vitro*, in normal human B lymphocytes (in which PUMA is barely detectable in the absence of such activation) (Figure 1A). This finding was confirmed *in vivo*: PUMA (mostly the 18 kDa Δ isoform) was found to be present in large amounts in germinal center B cells and their tumoral counterparts, Burkitt's lymphoma cells, such as BL41 cells (ref 15 and Figure 1B). Surprisingly, this significant increase in PUMA expression was found to be associated with cell proliferation, as assessed by thymidine uptake, rather than cell death (Figure 1A). This paradoxical observation suggested that the pro-apoptotic activity of PUMA was somehow inhibited, allowing the cells to grow and preventing them from dying. However, we previously observed that the amounts of Bcl-2, Bcl-XL and Mcl-1 were stable after mitogenic activation, indicating that the increase in PUMA expression was not counterbalanced by the production of large amounts of the anti-apoptotic counterparts of PUMA (see figure 1A in ref. 15). We checked that the protein observed was indeed PUMA, by assessing the specificity of the anti-PUMA antibody (Ab) targeting the C-terminal end of PUMA used in two ways: (i) Following immunoprecipitation of the FLAG-tagged recombinant α or β isoforms of PUMA with an anti-FLAG antibody, our anti-PUMA Ab was able to recognize both these isoforms (which were absent from the control Ig immunoprecipitate; Supplementary Figure 1A & 1B). (ii) Using two different siRNAs, we observed a knockdown of the expression of both the α (23 kDa) and β (18 kDa) isoforms of endogenous PUMA (Supplementary Figure 1C). The activity of some Bcl-2 family members is known to be regulated by their localization to particular compartments within the cell (e.g. Bax, Bid and Bad [16-18]). We therefore investigated the distribution of PUMA within these cells. Using a commercial kit (Calbiochem), we showed that PUMA was concentrated in the cytoplasmic fraction (fraction 1) of SAC-activated B cells. Almost no PUMA was found in the heavy membrane fraction (fraction 2), which included mitochondrion-associated proteins, such as VDAC, the BH3-only protein Bim and the anti-apoptotic protein Mcl-1. No PUMA was present in the fractions corresponding to the nucleus (fraction 3) or the less soluble fraction associated with the cytoskeleton (fraction 4) (Figure 1A). A similar distribution of PUMA and Bim was observed when BL41 cells were fractionated with the same kit (Figure 2A). For confirmation of these observations, we performed another type of fractionation, leading to the separation of cytosolic fractions (S25 or S) and mitochondrion-enriched fractions (P5 or P) (Figure 1C). Again, PUMA was present in the cytosolic fractions (S), whereas Bim and VDAC were present in the heavy membrane fractions (P) prepared from B41 cells (Figure 1B). PUMA was found in the cytosol of other BL cell lines, including CA46, Daudi and Ramos. EBV infection did not alter either the expression or the cytosolic localization of PUMA: no difference was observed between EBV-infected BL cells, such as BL41 95.8 or Ramos AW, and uninfected BL41 or Ramos cells (Figure 1D). As previously reported [19], Bim, unlike PUMA, displayed a strong downregulation of expression in the presence of EBV (Figure 1D). We then studied the cellular distribution of PUMA by immunofluorescence methods. Confocal microscopy showed that, unlike Mcl-1, PUMA did not colocalize with the mitochondria (TOM20). This finding confirmed that PUMA was cytosolic rather than located at the mitochondrial surface in BL41 cells (Figure 1E). Moreover, with the exception of the BH3-only protein Bid and the pro-apoptotic protein Bax, the cytoplasmic localization of which has been clearly established, the other Bcl-2 family members present in BL41 cells (the BH3-only proteins Bim, Noxa, Bik, the pro-apoptotic Bak and the anti-apoptotic proteins Mcl-1, Bcl-2 and BclxL) were all found in the mitochondrial fractions (Figure 1F).

Thus, PUMA had an unexpected, never before reported distribution in non-apoptotic activated human B cells and in Burkitt's lymphoma cells, in which it was localized to the cytosol.

### Apoptosis is associated with the translocation of PUMA to the mitochondria

We then investigated whether apoptotic signals affected the distribution of PUMA. The induction of BL41 cell apoptosis through BCR-mediated activation with cross-linked anti-μ Abs [20], drugs such as anisomycin [21] or by the UV treatment of HeLa cells [22] was associated with the translocation of PUMA from the cytosol to the mitochondria (Figure 2A, 2B, 2C and Supplementary Figure 2). This finding was confirmed by confocal microscopy, showing that anisomycin triggered the translocation of PUMA to the mitochondria, together with Bax expression in the mitochondria, and the release of cytochrome *c* into the cytosol (Supplementary Figure 2A & 2B). This suggests that the translocation of PUMA to the mitochondria may play a role in mitochondrial activation, leading to apoptosis. Consistent with this hypothesis, the translocation of PUMA to the mitochondria was also observed as early as 2 h after the exposure of HeLa cells to UV light, before appearance of apoptotic features, such as caspase-3 activation (manifested by PARP-1 cleavage) and cell shrinkage (Figure 2C). Furthermore, the siRNA-mediated downregulation of PUMA was correlated with the inhibition of anisomycin-mediated apoptosis in BL41 cells, strongly suggesting that this mitochondrial translocation of PUMA played an important role in the apoptotic process (Figure 2D). We checked that this translocation was directly associated with the mitochondrial apoptotic pathway, by activating HeLa cells with recombinant TRAIL. TRAIL-mediated apoptosis was not associated with the mitochondrial translocation of PUMA or the release of mitochondrial cytochrome *c* (Figure 2E panels a and b). These data provide strong support for our conclusion that PUMA translocation is associated with the mitochondrial apoptotic pathway. Nevertheless, it remains possible that the mitochondrion-associated PUMA was a newly synthesized protein rather than that localized to the cytosol in non-apoptotic cells. We addressed this question in two ways: (i) we quantified PUMA gene transcription, by carrying out qPCR on RNA isolated from BL41 cells activated with anisomycin or anti-μ antibody, or not activated. We observed no difference in PUMA mRNA levels between these cells (Supplementary Figure 3 panel a), (ii) we investigated the translocation of PUMA to the mitochondria in the presence of the protein synthesis inhibitor cycloheximide. We observed no decrease in the amount of PUMA in the mitochondria in the absence of protein synthesis (Supplementary Figure 3 panel b). Overall, these data fully support the conclusion that the PUMA present in the mitochondria came from the pre-existing cytosolic pool and was not synthesized *de novo*.

### PUMA overproduction is associated with mitochondrial localization and apoptosis

**Figure 3:**
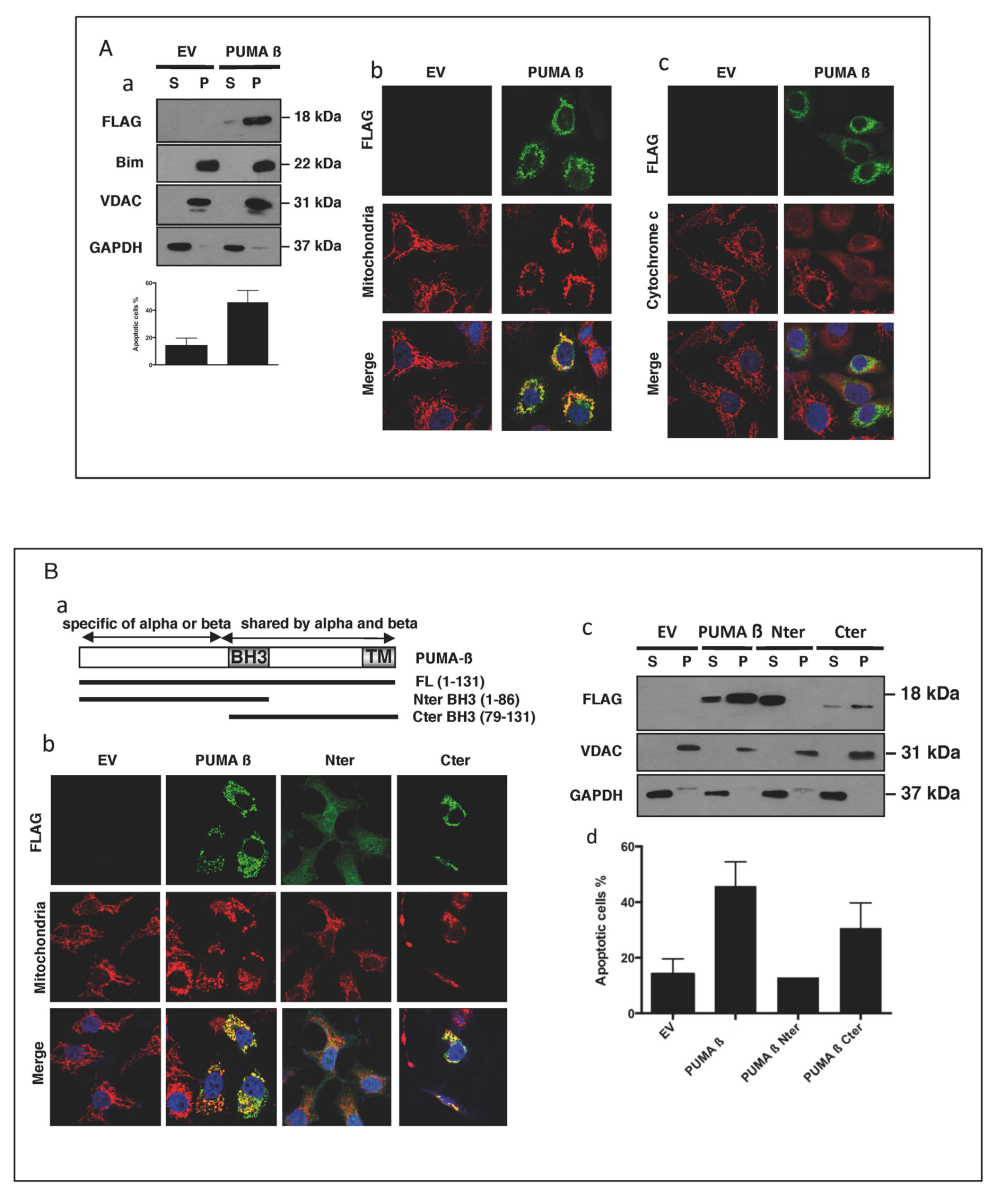
When overproduced, PUMA is found at the mitochondria and induces apoptosis **A.** HeLa cells, left untreated or treated for 30 min with 10 μM QVD-OPh, were transfected with a plasmid encoding a full-length Flag-tagged PUMA Δ (Puma Δ) or an empty vector (EV) and incubated for 24 h. The subcellular distributions of PUMA Δ, Bim, VDAC and GAPDH in QVD-OPh-treated cells were analyzed by western blotting (panel a). The data shown are means ± SD for triplicate experiments. EV- or PUMA Δ-transfected HeLa cells were stained with anti-FLAG and anti-HSP60 (panel b) or with anti-FLAG and anti-cytochrome *c* (panel c) primary antibodies, together with the corresponding fluorochrome-conjugated secondary antibodies, green for PUMA and red for HSP60 or cytochrome *c*. **B.** HeLa cells, treated for 30 min with QVD-OPh, were transfected with an empty vector (EV) or the full-length PUMA Δ (Puma Δ), N-terminal (Nter) or C-terminal (Cter) constructs of PUMA (panel a) and incubated for 24 h. Cells were stained with anti-FLAG and anti-HSP60 (mitochondria) primary antibodies, together with the corresponding fluorochrome-conjugated secondary antibodies, green for the various PUMA constructs and red for HSP60. Cytosolic (S) and mitochondrion-enriched (P) fractions were studied by western blotting (panel c), to determine the subcellular distribution of the proteins. HeLa cells were transfected with the various constructs, in the absence of QVD-OPh, incubated for 24 h and apoptosis was assessed by flow cytometry (panel c). Mean values ± SD for triplicate experiments are reported.

We overproduced PUMA in HeLa cells, to determine whether the translocation of PUMA to the mitochondria led to the induction of apoptosis. Both biochemical separation (Figure 3A, panel a) and confocal microscopy (panel b), showed that the overproduced PUMA was present at the mitochondria. This mitochondrial localization was associated with cytochrome *c* release (panel c) and cell shrinkage (panel a), suggesting that the presence of PUMA at the mitochondria was sufficient to induce cell death. We investigated the mechanisms underlying this translocation, by studying the localization and the pro-apoptotic activity of various truncated PUMA proteins. Constructs including the N-terminal or C-terminal ends of the β isoform of PUMA, including the BH3 domain, were overproduced in HeLa cells, and their cellular distribution was determined (Figure 3B panel a). The C-terminal fragment, like the full-length protein (PUMA β), was present at mitochondria (panels b and c), and this mitochondrial localization was associated with apoptosis (panel d). By contrast, the N-terminal fragment was found mostly in the cytosol of the transfected cells (Figure 3B panels b and c) and was not associated with cell death (panel d). We therefore verified that the N-terminal fragment was well produced in the presence (Figure 3B panel c) or the absence of the caspase-inhibitor Q-VD-OPh (Figure 3B panel d and Supplementary Figure 4 panel a). The data obtained showed that PUMA-mediated apoptosis was dependent on its mitochondrial localization and also implicated the C-terminal domain of PUMA in this localization. We also found that deletion of the BH3 domain of the C-terminal fragment abolished its apoptotic properties (Supplementary Figure 4 panel b). This suggests that, once translocated to the mitochondria, PUMA binds other Bcl-2 family proteins *via* its BH3 domain.

### Mitochondrial PUMA binds to and may inhibit Mcl-1 and Bcl-2 in BL41 cells

**Figure 4:**
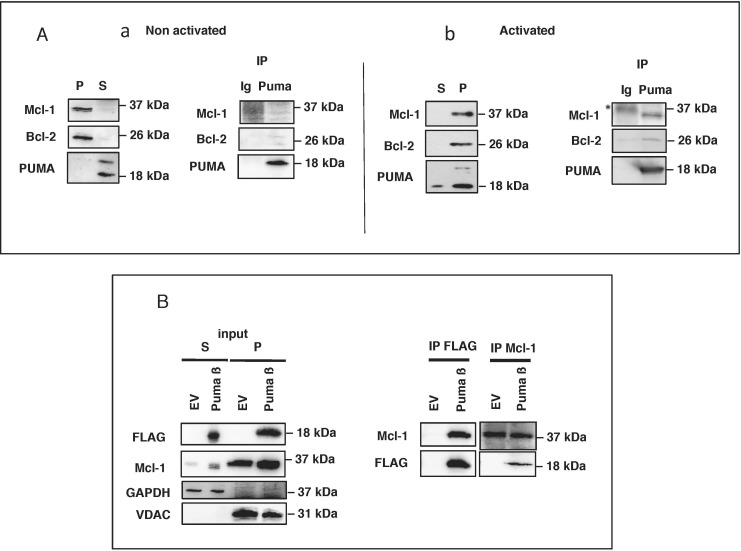
Mitochondrial PUMA binds to Mcl-1 and Bcl-2 in BL41 cells **A.** BL41 cells were left untreated or were treated for 4 h with anisomycin (2 μg/ml). Cell lysates were prepared and fractionated. S and P fractions from non-activated (panel a) and activated (panel b) cells were tested for Mcl-1, Bcl-2 and PUMA by western blotting. PUMA was immunoprecipitated (IP) from the S fraction of unstimulated cells (panel a) and the P fraction of stimulated cells (panel b); the immunoprecipitate was tested for Bcl-2 and Mcl-1 by western blotting (* non-specific band). **B.** HeLa cells pretreated with 10 μM QVD-OPh were transfected with an empty vector (EV) or a full-length FLAG-tagged PUMA construct (PUMA Δ), incubated for 24 h and lysed; cell lysates were separated into S and P fractions. The P fraction was subjected to IP with anti-FLAG or Mcl-1 antibodies. The resulting immune complexes were analyzed by western blotting with antibodies against PUMA (FLAG) or Mcl-1. VDAC and GAPDH were used as fraction purity controls for mitochondria and cytosol, respectively.

The apoptosis-mediating activity of PUMA, like that of all BH3-only proteins, is due to its binding, *via* its BH3 domain, with anti-apoptotic members of the Bcl-2 family (such as Mcl-1 or Bcl-2) and/or with the pro-apoptotic proteins Bax and Bak [9, 23-25]. We therefore investigated the association of PUMA with Mcl-1 and Bcl-2 in both activated and non-activated BL41 cells. No association between PUMA and these anti-apoptotic proteins (Figure 4A panel a) was observed in healthy cells, consistent with the cytosolic location of PUMA and the mitochondrial location of the anti-apoptotic proteins (Figure 1F). However, following activation with anisomycin, PUMA was translocated to the mitochondria, where it interacted with Mcl-1 and Bcl-2 (Figure 4A panel b). Likewise, the mitochondrial PUMA β recombinant protein, which strongly induced cell death (Figure 3A and 3B), was able to bind Mcl-1, as demonstrated by cross-immunoprecipitation of transfected cell lysates with anti-FLAG (PUMA) and anti-Mcl-1 Abs (Figure 4B). Unlike the full-length PUMA Δ and the C-terminal fragment, the N-terminal fragment, which was located in the cytosol, did not bind Mcl-1 (Supplementary Figure 4 panel c). Similar associations between PUMA and Bax or Bak proteins were not detected in our experimental conditions, either in activated BL41 cells or in transfected HeLa cells (data not shown). Nevertheless, both proteins seem to be required for the apoptotic properties of PUMA. Indeed, transfection of deficient MEF cells for Bax or Bak promotes an intermediate level of apoptosis in these cells whereas double deficient cells for both Bax and Bak were resistant to apoptosis mediated by PUMA β recombinant (Supplementary Figure 5). Altogether, these data show that (i) translocation of PUMA to mitochondria leads to its association with the anti-apoptotic proteins Mcl-1 and/or Bcl-2 and (ii) that apoptosis resulting from this translocation depends on both Bax and Bak.

### The translocation of PUMA to the mitochondria is caspase-independent but p38-dependent

**Figure 5:**
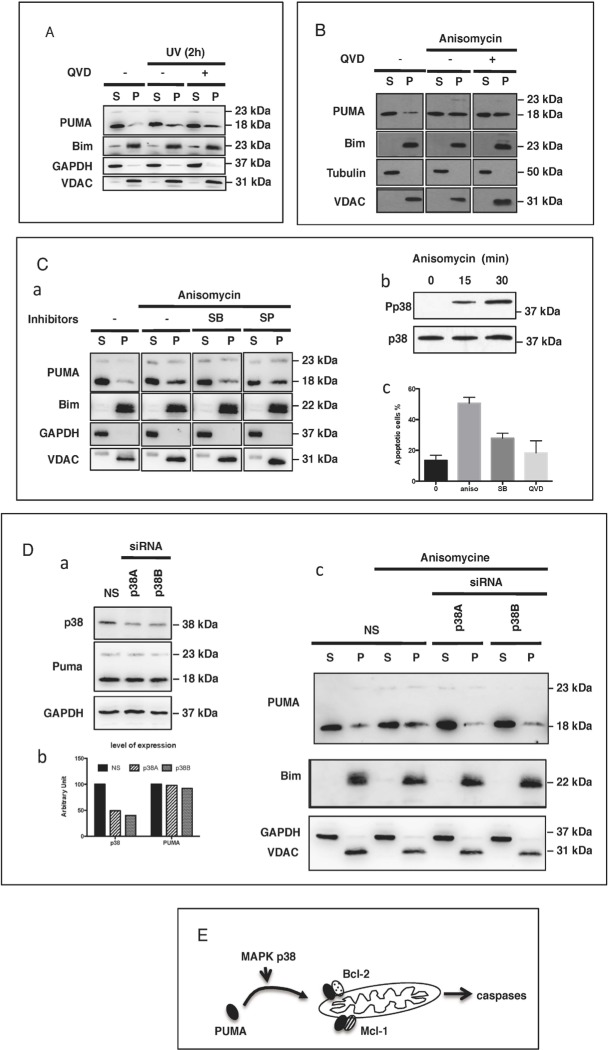
PUMA mitochondrial translocation (PMT) is caspase-independent but p38-dependent **A.** HeLa cells were treated or mock-treated with QVD-OPh (10 μM) for 30 min then exposed to UV (6 mJ/cm^2^) and cultured for an additional 2 h. The subcellular distribution of PUMA, Bim, GAPDH and VDAC was determined by subjecting western blotting the S and P fractions. **B.** BL41 cells treated or mock-treated with QVD-OPh (10 μM) were stimulated with anisomycin (2 μg/ml) for 4 h and the subcellular distributions of PUMA, Bim, tubulin and VDAC were determined by fractionation and western blotting. **C.** BL41 cells were treated or mock-treated with 10 μM SB203580 (SB) or 10 μM SP600125 (SP) for 30 min then stimulated for 4 h with anisomycin (2 μg/ml). The subcellular distributions of PUMA, cytochrome *c*, GAPDH and VDAC were analyzed by fractionation and western blotting (panel a). BL41 cells were treated with anisomycin (2 μg/ml) for 0, 15 or 30 min. Phosphorylated p38 levels were assessed by western blotting with an anti-phospho-p38 antibody (Pp38) and total p38 levels were assessed with an anti-p38 Ab (p38) (panel b). BL41 cells were treated or mock-treated with 10 μM SB203580 (SB) or 10 μM QVD-OPh for 30 min, then stimulated for 4 h with anisomycin (2 μg/ml), and apoptosis was assessed by flow cytometry (panel c). **D.** BL41 cells transfected with a non-targeting siRNA (NS) or a p38-targeting siRNA (p38A and p38B) for 76 h were treated for 0 (NS) or 4 h with anisomycin (2 μg/ml). The subcellular distributions of PUMA, Bim, GAPDH and VDAC were determined by western blotting the S and P fractions, and p38 knockdown efficiency was analyzed by western blotting with GAPDH as a loading control. **E.** Proposed model for the pathways regulating the translocation of PUMA to the mitochondria and role in apoptosis induction.

We assessed the dependence of PUMA translocation on caspases, given the key role that caspases are known to play in apoptotic cell death. Prior treatment of BL41 cells with the pan-caspase inhibitor Q-VD-OPh did not prevent the mitochondrial translocation of PUMA following UV exposure (Figure 5A) or treatment with apoptotic doses of anisomycin (Figure 5B). These findings suggest that caspase activation occurs downstream from the translocation of PUMA to the mitochondria. Various members of the MAPK family of kinases play a major role in early signaling. We investigated their possible role in controlling the translocation of PUMA to the mitochondria. We found that, following anisomycin treatment, the translocation of PUMA to the mitochondria was significantly decreased by the MAPK p38 inhibitor SB203580, whereas a more specific inhibitor of JNK (SP600125) had only a very weak effect (Figure 5C panel a). As a control, we checked that anisomycin promoted the phosphorylation and activation of p38 in our experimental conditions (Figure 5C panel b) and that the inhibition of p38 decreased anisomycin-mediated apoptosis (Figure 5C panel c). Furthermore, the siRNA-mediated downregulation of p38 was associated with inhibition of the anisomycin-induced translocation of PUMA to the mitochondria (Figure 5D). We also observed a very similar pattern in HeLa cells treated with UV: (i) p38 was phosphorylated (Supplementary Fig. 6 panel a) and (ii) and the decrease of p38 expression with specific siRNA (Supplementary Figure 6 panel b) inhibited the mitochondrial translocation of PUMA (Supplementary Figure 6 panel c). In this context, no change was observed in the levels of anti-apoptotic proteins, such as Bcl-XL which is present in large amounts in HeLa cells (Supplementary Figure 6 panel d). These findings are consistent with the notion that apoptotic stimuli (such as anisomycin or UV treatment) can induce the translocation of cytosolic PUMA to the mitochondria, in a p38-dependent manner. After reaching the mitochondria, PUMA can bind to and inhibit Mcl-1 and/or Bcl-2, thereby promoting mitochondrial activation and the subsequent activation of caspases and apoptosis (Figure 5E).

## DISCUSSION

We describe here an original mechanism controlling PUMA activation: its translocation from the cytosol to the mitochondria. The pro-apoptotic activity of PUMA is believed to be largely controlled by the transcriptional and posttranslational regulation of its expression, through the modulation of its degradation. The general consensus is that once produced, PUMA is targeted to the mitochondria, where it can act as a pro-apoptotic protein [26], as observed in our experimental conditions after the synthesis of recombinant PUMA, eventually inducing cell death. However, in some conditions, the upregulation of PUMA may be associated with cell activation and proliferation rather than apoptosis. This situation has been observed, for example, during the activation of human B lymphocytes *in vitro* following stimulation of the BCR and CD40 receptor, or activation with mitogens, such as SAC [15]. *In vivo*, germinal center B cells and Burkitt's lymphoma cells (their tumoral counterpart) also produce large amounts of PUMA without the occurrence of cell death [15]. We show here that the role of PUMA in these non-apoptotic cells is dependent on its cytosolic localization. Our results also demonstrate that the translocation of PUMA from the cytosol to the mitochondria, in the absence of transcriptional regulation, is an alternative pathway for the expression of the pro-apoptotic activity of PUMA, at least in some cell types, including B lymphocytes. Our findings indicate that, in our system, PUMA must be “activated” by translocation to the mitochondria, to induce apoptosis. Several unanswered questions remain about the maintenance of PUMA in the cytosol. Other BH-3-only proteins are located in the cytoplasm or associated with the cytoskeleton. Bim and Bmf can be associated with skeleton structures, such as tubulin and actin [27, 28], respectively, whereas other proteins are sequestered in the cytoplasm through direct interaction with the chaperone protein 14-3-3 (Bad) or through interactions with unknown partners or in the absence of partners, as for Bid [18, 29, 30]. The subcellular distribution of endogenous PUMA has not been extensively studied. PUMA is generally described as mitochondrial, but one group reported interactions between PUMA and the cytosolic domain of the activated EGFR and EGFRvIII proteins, resulting in its cytosolic sequestration in glioblastoma cells [31]. We detected no interactions, in our cells, between PUMA and other members of the ErbB family (EGFR is not expressed by B cells), such as ErbB1, ErbB2, ErbB3 or ErbB4, which may be present at various levels in some B cells, such as multiple myeloma cells [32]. We have not yet identified any putative cytosolic partners of PUMA in B cells, although mass spectrometry analysis has suggested various potential candidates for further investigation. Alternatively p38 may mediate an increase in PUMA transcription, by activating p53, with or without c-Abl [33-35]. It therefore remains possible that the PUMA in the mitochondria was synthesized *de novo* rather than being translocated from the cytosol of non-apoptotic cells. However, this seems unlikely in our model, because (i) we observed no increase in PUMA mRNA levels in our experimental conditions and (ii) the mitochondrial translocation of PUMA was not prevented by the inhibition of protein synthesis.

Our data indicate that the C-terminal domain, rather than the BH3 domain, is crucial for the targeting of PUMA to mitochondria. Indeed, the N-terminal fragment containing the BH3 domain was located in the cytosol, whereas the C-terminal fragment was associated with mitochondria and able to induce the death of transfected cells. The BH3 domain is therefore not sufficient to target PUMA to mitochondria, whereas the C-terminal domain common to the α and β isoforms of PUMA and containing a putative transmembrane domain, is necessary for the mitochondrial localization of the protein [36]. These findings suggest that the mitochondrial localization of PUMA involves the insertion of PUMA into the mitochondrial outer membrane rather than simple contact with mitochondrial outer membrane proteins [37]. Three issues need to be addressed: (i) the molecular structure of cytosolic PUMA, (ii) the molecular basis of its translocation to the mitochondria and (iii) the nature of the mitochondrial partners of PUMA.

Phosphorylation regulates the activity of many proteins. The MAPK family of kinases plays an important role in regulating the expression and localization of various BH3-only proteins. Indeed, Erk can phosphorylate Bim, driving its degradation by the proteasome [38] [20]. Phosphorylation by JNK may control the release of Bim and Bmf from tubulin and actin, respectively, and JNK-mediated Bid phosphorylation prevents Bid cleavage by caspase-8 and, thus, its activation [27, 28, 39-41]. Phosphorylation have been reported to affect Bim and PUMA degradation [20, 42, 43]. Sandow et al. recently reported that IKK-mediated phosphorylation of the PUMA S10 residue can induce the degradation of this protein by the proteasome [44]. Interestingly, the β isoform, major isoform present in human B cells ([15] [21] and this paper), lacks the S10 residue, suggesting other residues may be involved in a similar way. Indeed, Fricker et al. described also other phosphorylation sites besides S10 (the predominant one) whose effect on PUMA expression or function remains unknown [43]. It has also been suggested that p38 may trigger an increase in PUMA transcription *via* p53 (with or without c-Abl activation ([33] [35] [34]). This suggests that the PUMA associated with mitochondria might be newly synthesized rather than translocated from the cytoplasm. This is unlikely in our model because (i) there was no increase in PUMA mRNA levels in our experimental conditions and (ii) the translocation of PUMA to the mitochondria was observed in the presence of the protein synthesis cycloheximide. Thus, the translocation of PUMA to the mitochondria is dependent on p38 activation. We are therefore currently trying to determine whether (i) PUMA is phosphorylated upon apoptotic stimuli and (ii) a direct phosphorylation of PUMA by p38 is required for its translocation, and, if not, which targets of p38 might be involved

Once translocated to the mitochondria, PUMA can associate with other members of the Bcl-2 family [26]. Our data clearly show that, following apoptotic stimulation, PUMA can bind to and potentially inhibit both Mcl-1 and Bcl-2 in BL41 cells. This finding is consistent with previous demonstrations that the pro-apoptotic activity of PUMA depends on its capacity to interact with, and thereby inhibit the biologic activity of anti-apoptotic proteins of the Bcl-2 family, such as Mcl-1 and Bcl-2. However, there is evidence that PUMA may also associate with and directly activate the pro-apoptotic molecules Bax and Bak [23, 26, 45], although this remains to be demonstrated definitively. In our experimental conditions, we detected no direct association between PUMA and Bax or Bak. This implies that (i) no such association occurred in our experimental conditions or, more probably, (ii) no such association was detectable in the physiological conditions used (almost all the experiments showing such an interaction were performed with recombinant proteins) [23, 45, 46]. This is consistent with the “hit and run” model involving a transient interaction of PUMA with Bax and Bak [47-49]. Nevertheless, our data show that PUMA-mediated apoptosis is directly dependent on the production and function of both Bax and Bak, because, in the absence of these molecules, the mitochondrial translocation of PUMA does not lead to apoptosis.

PUMA, along with Bid and Bim, is a major regulator of apoptosis involved in the responses of various types of cells to a large panel of apoptotic stimuli. It is therefore important to determine whether the original mechanism observed in Burkitt's cells is also found in other cell types. We also observed the same cytosolic localization of PUMA in various multiple myeloma cell lines, the tumoral counterpart of normal plasma cells of the B-cell lineage (data not shown). We are currently studying the distribution of PUMA in a wide variety of tumor cells, to determine whether the presence of PUMA in the cytoplasm is restricted to lymphoid cells or is a more general feature as suggested by our observations in HeLa cells. Our data suggest that the accumulation of PUMA in the cytosol may be an important feature in the differentiation and tumorigenesis processes, allowing this protein to participate in the apoptotic process without the need for additional transcription.

## MATERIALS AND METHODS

### Antibodies and reagents

We used primary antibodies against the following proteins for immunoblotting (IB) and immunofluorescence staining (IF): PUMA (C-Term, 1652-1, clone EP512Y) and Bim (1036-1, clone Y36) from Abcam; Bax (N-20, sc-493), Bcl-2 (C-2, sc-7382), Bcl-XL (S18, sc-634), Bik (FC-160, sc-10770), HSP60 (N-20, sc-1052), Mcl-1 (S-19, sc-819), Tom20 (F-10, sc-17764), and tubulin (TU-02, sc-8035) from Santa Cruz Biotechnology; VDAC (AB10527) from Merck Millipore; cytochrome *c* (IB: 556433; IF: 556432) and PARP-1 (556362, clone C2-10) from BD Pharmingen; FLAG (F1804, clone M2) and GAPDH (G9545) from Sigma-Aldrich; NOXA (IMG-349A) from Imgenex; Bid (AF860) from R&D Systems; Bak (38145) caspase-3 (9662) and P-p38 (9211) from Cell Signaling Technology.

Anti-human IgM (DA.44) monoclonal antibodies from the American Type Culture Collection (ATCC) were purified on protein A-Sepharose columns from Pharmacia Biotech. Anti-mouse IgM antibody was obtained from Jackson Immunotech.

The following reagents were used: anisomycin (A9789) and cycloheximide from Sigma-Aldrich; Q-VD-OPh (OPH001) and recombinant TRAIL from R&D Systems; N-tosyl-Phe chloromethyl ketone (TPCK, 616387) and SP600125 (420119) from Calbiochem and SB203580 (PHZ1253) from Life Technologies.

### RNA interference

PUMA/BBC3 silencing was achieved with two small interfering RNAs (siRNA), BBC3HSS146895 and BBC3HS178326 referred to as “P1” and “P2” respectively. Medium GC duplex (12935-112) was used as a negative control. All these siRNA oligomers were purchased from Life Technologies. P38 silencing was achieved with 2 siRNAs, SASI_Hs01_00018465 and SASI_Hs01_00018464, referred to as “p38A” and “p38B” respectively, from Sigma-Aldrich. We assessed siRNA knockdown efficiency by western blotting.

### Plasmid construction

The full-length cDNA for the human Δ isoform of PUMA (kindly provided by Dr P. Juin, Nantes) was inserted into p3XFLAG-CMV-10 (Sigma-Aldrich, E7658). The sequences encoding N-terminal-BH3 (1-258) and C-terminal-BH3 (232-395) were amplified by PCR and ligated into the same plasmid. All constructs were checked by DNA sequencing.

### Cell culture and DNA and siRNA transfections

The BL41, BL41 95.8, Ramos, Ramos AW, DAUDI and CA46 cell lines were cultured in RPMI 1640 medium with GlutaMAX^™^, supplemented with 10% heat-inactivated fetal bovine serum (FBS), 10 mM HEPES, 100 U/ml penicillin, 100 μg/ml streptomycin, 1 mM sodium pyruvate and 1 x nonessential amino acids (Life Technologies). HeLa and MEF cells were cultured in DMEM (Sigma, D0819) supplemented with 10% heat-inactivated FBS, 0.1 mg/ml Normocin^™^ (InvivoGen) and antibiotics. SV-40-transformed Bax^−/−^, Bak^−/−^, DKO and the corresponding wild-type (WT) MEF cells were generously provided by Stanley J Korsmeyer (Harvard Medical School, Boston, MA, USA).

BL41 cells were transfected with siRNA in the BTX-Harvard Apparatus ECM 830 Square Wave Electroporation System (Fisher Scientific). For each siRNA, 4×10^6^ BL41 cells were washed twice in RPMI 1640 without GlutaMAX^™^ and resuspended in 400 μl of medium in a cuvette, with 80 nmol of the appropriate siRNA. Cells were then electroporated at 240 V for 10 ms, and transferred to six-well plates in a final volume of 4 ml. The experiments were carried out 72 h later.

HeLa cells were transiently transfected with empty and PUMA-encoding p-CMV-neo vectors, with Fugene HD (Promega, E2311), according to the manufacturer's protocol or with 5 mol of the appropriate siRNA, with oligofectamine (Invitrogen, 12252-011). Cells were treated with Q-VD-OPh for 30 min, then transfected with DNA.

### qPCR

RNA was extracted from the cultured cells with the RNeasy Plus mini kit (Qiagen), according to the manufacturer's protocol. Cell lysates for RNA extraction were obtained by homogenization with QIAshredder spin columns (Qiagen). We synthesized cDNA with the RevertAid H Minus First Strand cDNA Synthesis Kit (Thermo Scientific). In brief, 5 μg RNA was combined with oligo (dT)_18_ primer (10 μM), H_2_O, 5x reaction buffer, 10 mM dNTP mix, RiboLock RNase inhibitor (20 U/μL) and RevertAid H Minus M-MuLV reverse transcriptase (200 U/μL). The mixture was incubated at 42°C for 60 minutes and then at 70°C for five minutes to stop the reaction. We carried out qPCR in a volume of 20 μl containing 5 μl undiluted cDNA, primers (0.75 μM final concentration) and Power SYBR^®^ Green Master Mix (Applied Biosystems). The following primers were used: PUMA (fw 5′-GACGACCTCAACGCACAGTA-3′; rv 5′- CTAATTGGGCTCCATCTCG-3′)* and 18s (fw 5′-AGAAACGGCTACCACATCCA-3′; rv 5′-CACCAGACTTGCCCTCCA-3′)** (SIGMA). PCR was performed on an Mx3005P qPCR System (Agilent Technologies), as follows: 95°C for 10 min followed by 40 cycles of 95°C for 15 s, 60°C for 30 s, 72°C for 30 s. PCR efficiency was determined with 10-fold dilutions, as follows: Efficiency **E.** = 10^(−1/slope)^. The ratio of PUMA expression in treated cells to PUMA expression in untreated cells was determined as follows: Ratio = ((E_PUMA_)^ΔCt PUMA (control-treated)^)/((E_18s_)^ΔCt 18s (control-treated)^).

### Immunofluorescence (IF) staining and confocal microscopy

Cells were fixed by incubation with 4% paraformaldehyde (Alfa Aesar, 43368) for 15 min, then permeabilized by incubation with 0.15% Triton-X100 (Sigma-Aldrich, X100) for 15 min. Samples were blocked by incubation with 2% BSA (Sigma, A9576) for 30 min. The cells were incubated with relevant primary antibodies at a dilution of 1:400 for 1 h at room temperature or overnight at 4°C, then with the corresponding secondary antibodies conjugated with fluorescent dyes (Alexa Fluor^®^, Life Technologies), at 1:800, for 1 h at room temperature, in the dark. DAPI (Life Technologies, D1306) was then incubated with the cells, at a dilution of 1:10,000 for five minutes, for nuclear staining and cells were mounted on cover slips in 7 μL of Fluoromount-G^™^ slide mounting medium (Beckman Coulter, 731604). Cells were washed three times with PBS between steps. Images were acquired with a Leica SP5 confocal microscope (Leica Microsystems) equipped with a x63 oil immersion fluorescence objective.

### Western blot analysis

Whole-cell lysates were prepared by incubation of the cells with TNT buffer (50 mM Tris-HCl, pH 7.4, 150 mM NaCl, 2 mM EDTA, 1% Triton and 1% Igepal/NP-40), supplemented with Halt^™^ protease inhibitor cocktail (Thermo Scientific, 1861279) for 30 min. The lysates were clarified by centrifugation at 10,000 x *g* for 10 min. Cells were fractionated with the ProteoExtract Subcellular Proteome Extraction Kit (Calbiochem, 539790), according to the manufacturer's protocol. At least 4×10^6^ cells were lysed mechanically in 27G 1/2 Tuberculin syringes (Sigma, Z192082-100EA), in 200 μL of H60 buffer (20 mM HEPES, 1.5 mM MgCl_2_, 60 mM KCl) supplemented with the same protease inhibitor cocktail (18 back and forth movements), and fractionated by differential centrifugation (procedure detailed in Figure 1C). The P5 pellets were washed once in H60 buffer and lysed in TNT buffer with 1% SDS for 30 min. Protein quantity was measured with the microBCA protein assay kit (Thermo Scientific, 23235). Protein samples (usually 5 μg) were then boiled for 5 min at 99°C after the addition of Tris-Glycine SDS sample buffer (Life Technologies, LC2676) with 10% Δ-mercaptoethanol (Sigma, M3148), separated on gradient 5-20% polyacrylamide gels and transferred to nitrocellulose membranes (Santa Cruz, sc-201698). The membranes were incubated with specific antibodies, and bound antibody was visualized by chemiluminescence with the Immobilon western chemiluminescence HRP substrate (Millipore, WBKLS0500) and a DDC camera (LAS-4000 mini, Fujifilm).

### Immunoprecipitation

500 μl of whole cell lysates, cytosol fractions (S25) or mitochondria-enriched fractions (P5) were pre-cleared by adding 40 μl of protein G-sepharose beads (Sigma-Aldrich, P3296) and placed on a spinning wheel at 4°C. After 30 min, the beads were pulled down by centrifugation at 15,000g for 10 sec. The supernatant was collected and 2 μg of relevant antibody and 40 μl of protein G-sepharose beads were added, and the samples were spinned at 4°C for 2h. The beads were pulled down by centrifugation, and the supernatant discarded. Bound proteins were eluted from the beads with 30 μl of Tris-Glycine SDS sample buffer with 10% Δ-mercaptoethanol. Samples were then analyzed by western blotting.

### Detection of apoptotic cells

The dot-plot scatter profiles of the cells were analyzed by flow cytometry with a BD Accuri C6 Flow Cytometer^®^ (BD Biosciences). Shrunken cells with high side scatter (SSC) and low forward scatter (FSC) were considered apoptotic. The number of apoptotic cells is expressed as a percentage of the total population.

## SUPPLEMENTARY MATERIAL FIGURES


