# Supplementary material for: Subcellular localization of PUMA regulates its pro-apoptotic activity in Burkitt's lymphoma B cells
Source: Oncotarget. 2015 Sep 29;6(35):38181–94. doi: 10.18632/oncotarget.5901 (PMC4741992; doi:10.18632/oncotarget.5901)
Supplement: Supplementary file 1 [file oncotarget-06-38181-s001.pdf]

# Subcellular localization of PUMA regulates its pro-apoptotic activity in Burkitt's lymphoma B cells

**Supplementary Material**

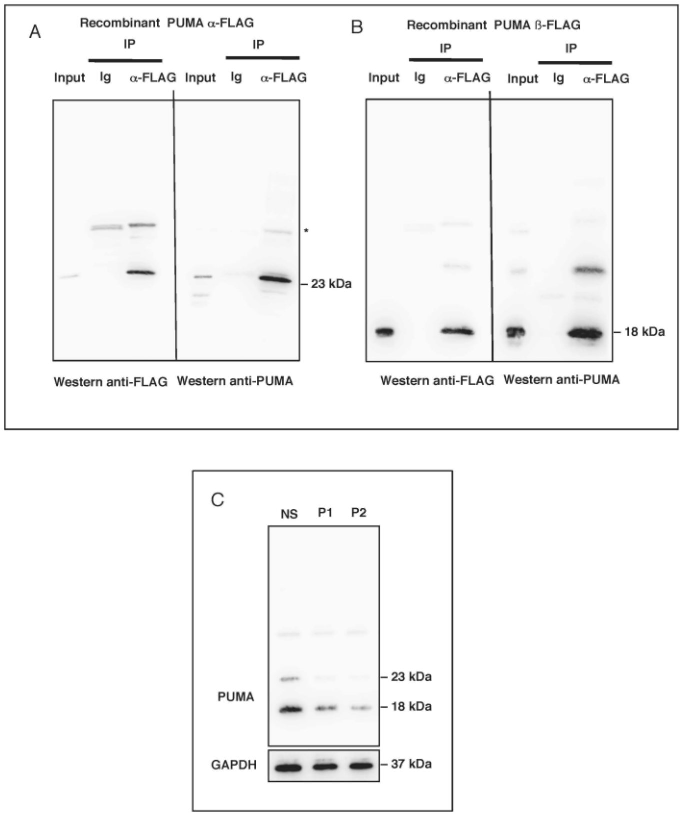


**Supplementary Figure 1**: The anti-PUMA Ab **(**clone EP512Y from Abcam) recognize both α and ß isoforms of PUMA.

Recombinant FLAG-tagged PUMA ß (**A**) or FLAG-tagged PUMA α (**B**) proteins were immunoprecipitated with the anti-FLAG Ab. The immunoprecipitates were analyzed for the presence of PUMA α (23 kDa) or PUMA ß (18 kDa) by western blotting with anti-FLAG or anti-PUMA (clone EP512Y from Abcam) Abs. (* non-specific band). BL41 cells were transfected with a non-targeting siRNA (NS) or PUMA-targeting siRNA (P1 and P2) for 76 h and PUMA levels were assessed by western blotting with the anti-PUMA Ab **(**clone EP512Y).

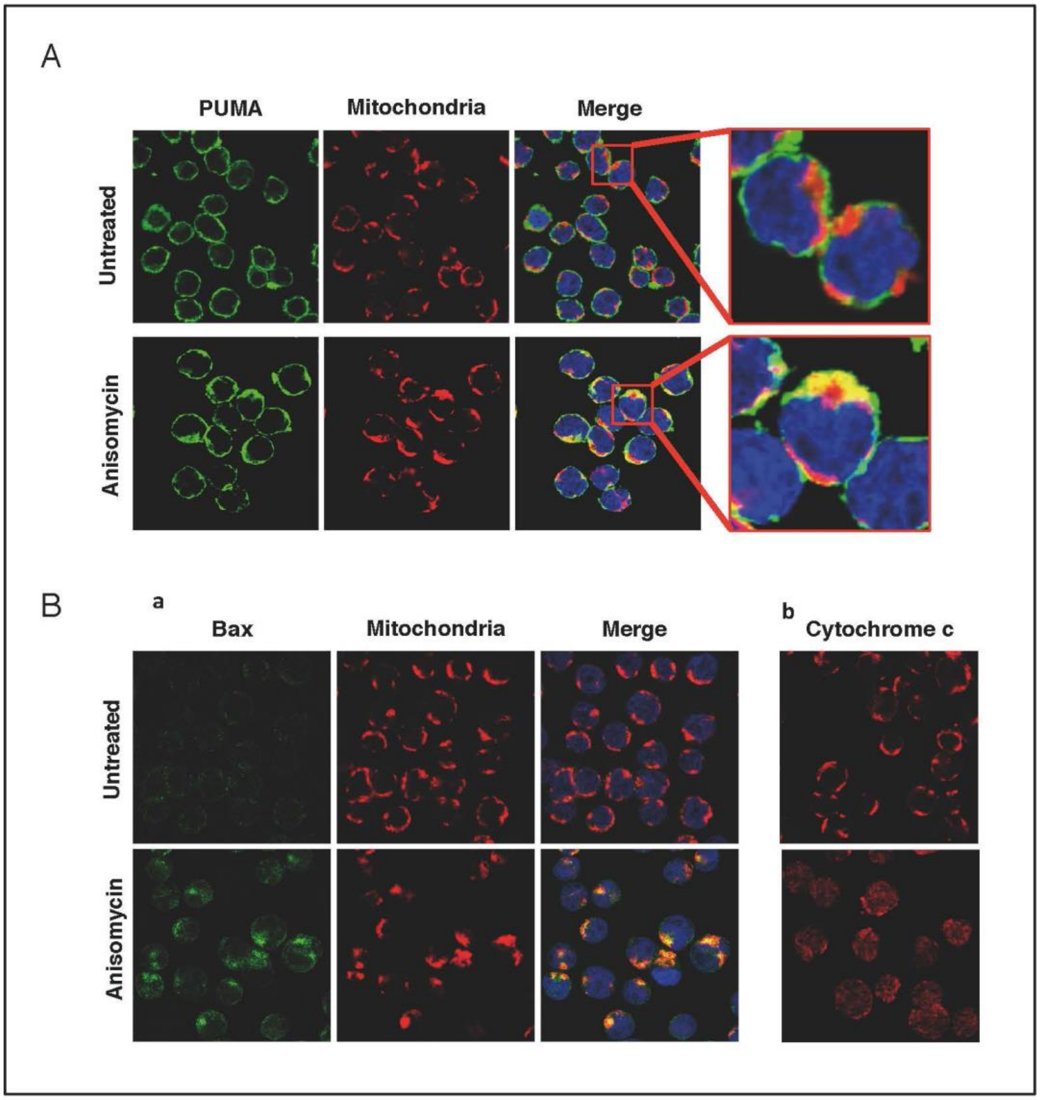


**Supplementary Figure 2**: PUMA translocates to the mitochondria upon anisomycin-induced apoptosis.

BL41 cells were stimulated with anisomycin (2 µg/ml) for 0 or 4 h, and then stained with anti-PUMA (**A**) or anti-Bax (**B: a**) and anti-TOM20 primary antibodies, together with the corresponding fluorochrome-conjugated secondary antibodies, green for PUMA and Bax or red for TOM20, for analysis of the subcellular distributions of PUMA and Bax by confocal microscopy. (**B: b**) BL41 cells were stimulated as in A and B and stained with anti-cytochrome *c* primary antibodies and secondary antibodies conjugated to a red fluorochrome, to analyze cytochrome *c* release, the hallmark of mitochondrial apoptosis.

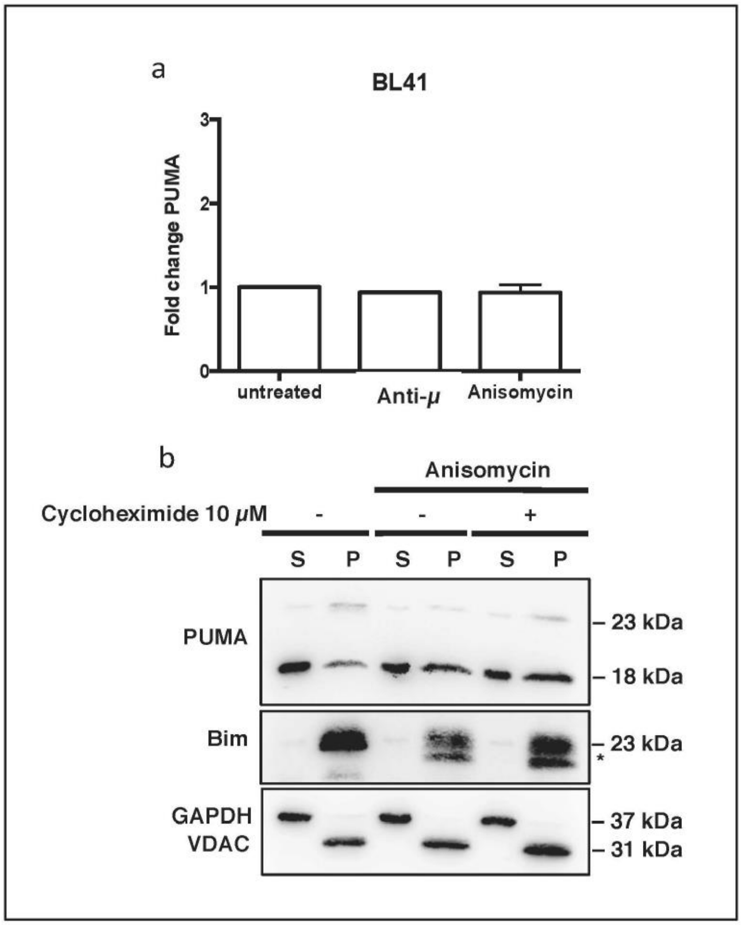


**Supplementary Figure 3:** Mitochondrial translocation of PUMA is not dependent o de novo protein synthesis.

BL41 cells were stimulated with mouse anti-human μ antibodies (5 μg/ml) cross-linked with anti-mouse IgM antibodies (28 μg/ml) or anisomycin (2 μg/ml) and PUMA mRNA levels were quantified by qPCR (panel a). Mean values +/- S.D. of three different experiments. BL41 cells were treated or mock-treated with 10 μM cycloheximide for 30 min, followed by anisomycin (2 μg/ml) for 4 h, and the subcellular distributions of PUMA, Bim, GAPDH and VDAC were determined by western blotting the S and P fractions.

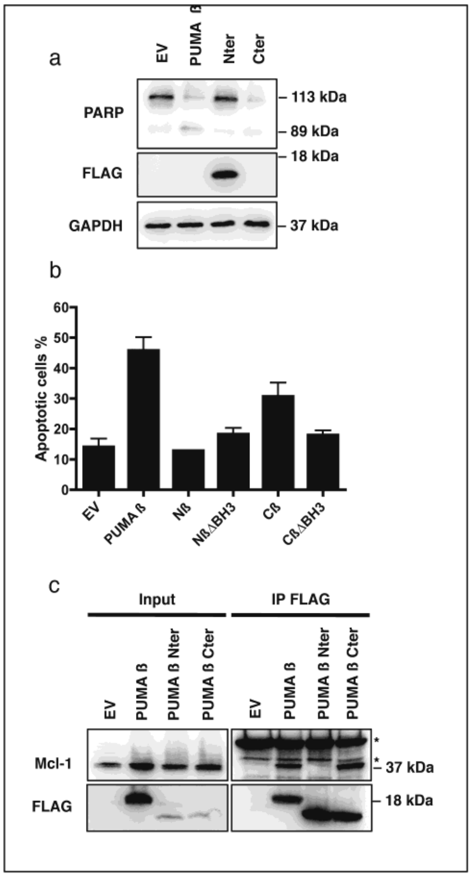


**Supplementary Figure 4:** N-ter and C-ter PUMA differ in their apoptotic properties.

**(panel a)** HeLa cells were transfected with an empty vector (EV) or full-length PUMA ß (Puma ß), N-terminal (Nter) or C-terminal (Cter) constructs of PUMA (panel a) and incubated for 24 h. PARP-1 cleavage and recombinant protein production were assessed by western blotting with anti-PARP anti-FLAG Abs, respectively. (**panel b**) HeLa cells were transfected with an empty vector (EV) or full-length PUMA ß (Puma ß), N-terminal (Nß), BH3-deleted N-terminal (NßΔBH3), C-terminal (Cter) or BH3-deleted C-terminal (CßΔBH3) constructs of PUMA (panel a) and incubated for 24 h. Apoptosis was assessed by flow cytometry. Mean values ± SD of triplicate experiments are reported. (**panel c**) HeLa cells were transfected with an empty vector (EV) or full-length PUMA ß (Puma ß), N-terminal (Nter) or C-terminal (Cter) constructs of PUMA (panel a) and incubated for 24 h. Cell lysates were subjected to IP with an anti-FLAG antibody. The resulting immune complexes were analyzed by western blotting with antibodies against PUMA (FLAG) or Mcl-1.

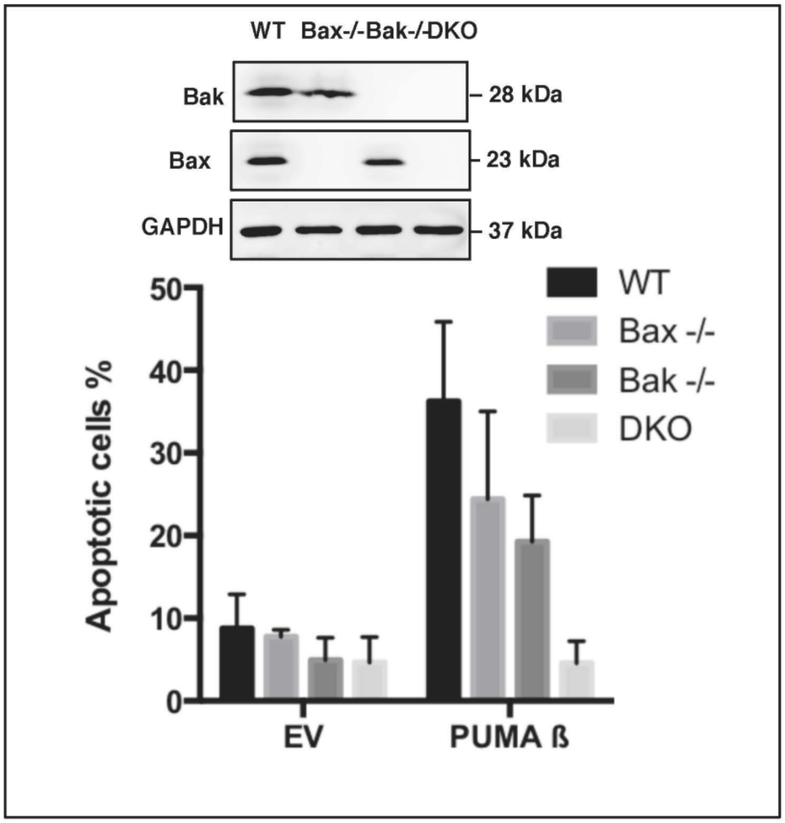


**Supplementary Figure 5:** PUMA-mediated apoptosis is dependent of Bax and Bak expression.

Bax-/-, Bak-/- or Double Knock Out (DKO, Bax-/- Bak-/-) MEF cells and their wild-type (WT) counterparts were transfected with an EV or Puma ß vector, incubated for 24h, and apoptosis was assessed by flow cytometry. The values reported are means +/- S.D. of triplicate experiments. Bak, Bax and GAPDH expression, analyzed by western blotting, in the three cell lines is shown in the inset.

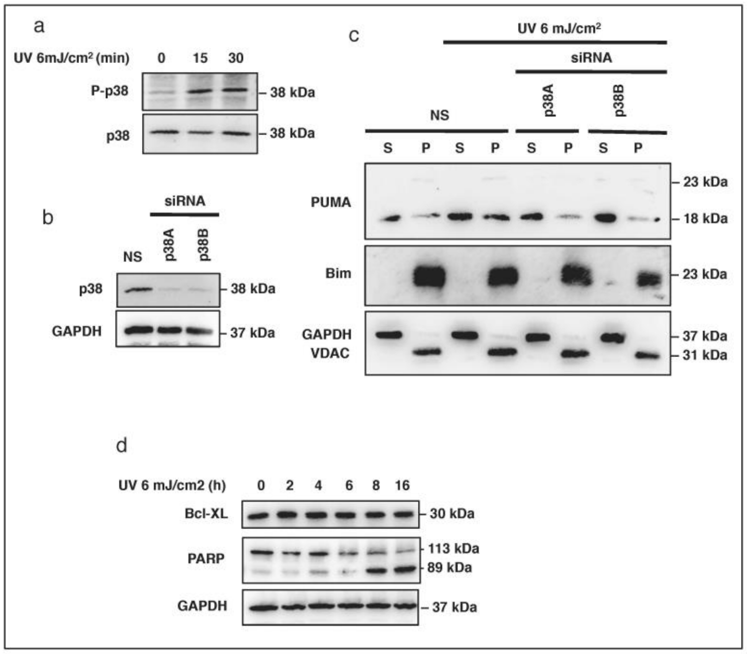


***Supplementary Figure 6:*** *UV-mediated mitochondrial translocation of PUMA is 38 dependent.*

(**panel a**) HeLa cells were exposed to UV (6 mJ/cm$^2$) and cultured for 0, 15 or 30 min. Phosphorylated (Pp38) and total p38 were analyzed by western blotting. (**panel b**) Hela cells were transfected with a non-targeting siRNA (NS) or a p38-targeting siRNA (p38A and p38B) for 48h and p38 expression was assessed by western blotting. GADPH was used as a loading control. (**panel c**) Hela cells were transfected with a non-targeting siRNA (NS) or a p38-targeting siRNA (p38A or p38B) for 48h, subjected to UV exposure (6 mJ/cm2) and then left in culture for 2h. The subcellular localization of Puma, Bim, GAPDH and VDAC was determined by subjecting S and P fractions to western blotting. (**panel d**). HeLa cells were exposed to UV (6 mJ/cm2) and cultured for the indicated time. Bcl-XL expression and PARP-1 cleavage were assessed by western blotting.
